# Supplementary material for: Cytoskeletal Responses and Aif-1 Expression in Caco-2 Monolayers Exposed to Phorbol-12-Myristate-13-Acetate and Carnosine
Source: Biology (Basel). 2022 Dec 25;12(1):36. doi: 10.3390/biology12010036 (PMC9855102; doi:10.3390/biology12010036)
Supplement: Supplementary file 1 [file biology-12-00036-s001.zip › biology-2032174-supplementary.pdf]

Image of WB for beta-actin as reported in results

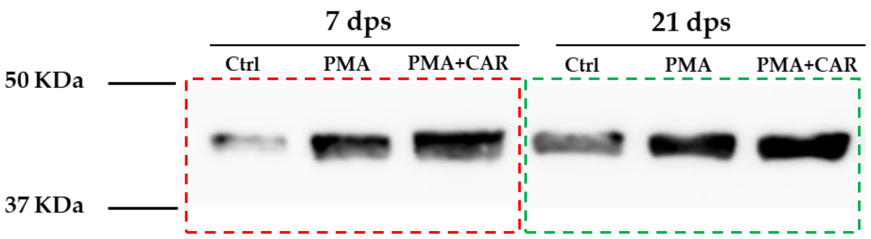

Precision Plus Protein All Blue Standards

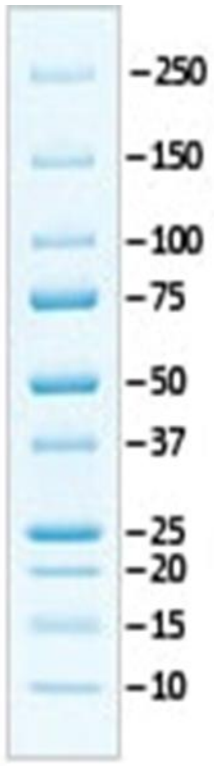

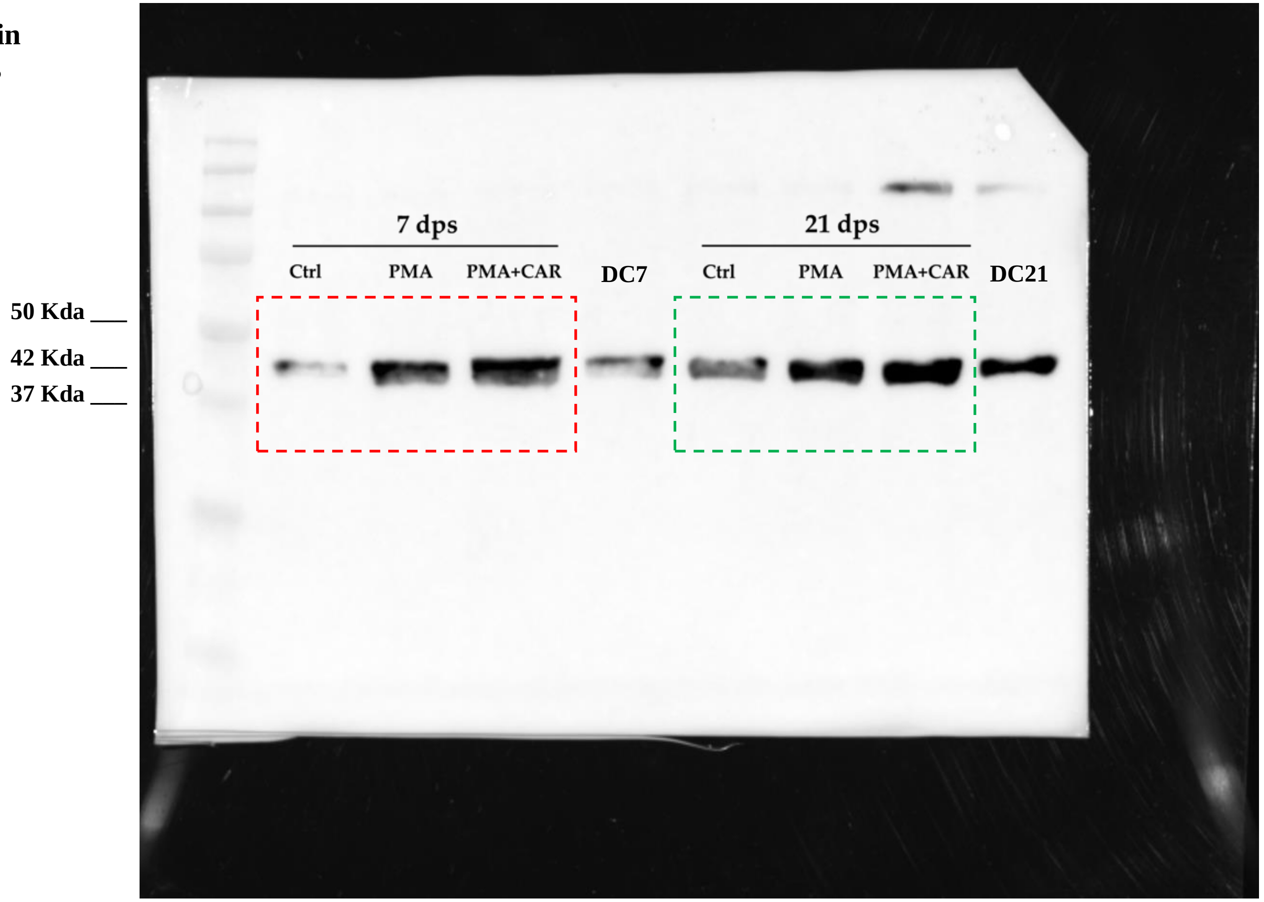

DC7/21: doubled control at 7/21 dps

20 ug protein loading per lane

Western blotting details:

Following electrophoresis on stain-free pre-cast 12% polyacrylamide gels (Bio-Rad), proteins from cell lysates were electro-transferred to polyvinylidene fluoride (PVDF) membranes. After protein transfer, the membranes were blocked for 5 minutes in EveryBlot Blocking Buffer (Bio-Rad), and then immunoblotted in EveryBlot Blocking Buffer with the following primary antibody: **mouse monoclonal anti-β-actin primary antibody** (Sigma-Aldrich, clone AC-15, product ID A5441; dilution 1:20000). After rinsing, blots were incubated with conjugated anti-mouse secondary antibody (Sigma-Aldrich) and the immune reactive bands were detected using an enhanced chemiluminescence method (ECL kit, Bio-Rad) with ChemiDocTM Imaging System (Bio-Rad). Densitometric quantitative analysis was carried out by using the Image Lab software version 6.1 (Bio-Rad). The pixel intensity for each band was analyzed, the background was subtracted, and the protein expression results were normalized with respect to the total lane of the loaded samples. As molecular weight markers, the Precision Plus Protein All Blue Standards (Bio-Rad, cat. no. #161-0373) was used.

Image of WB for beta-actin as reported in results

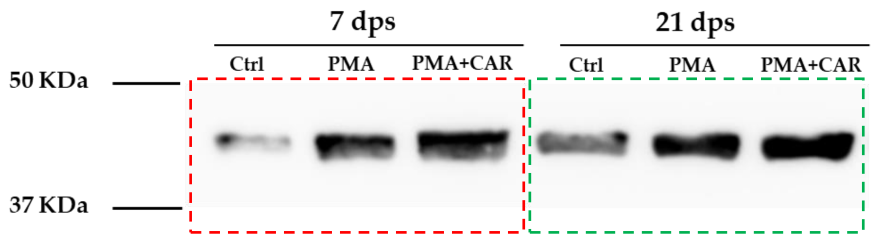

	biological replicates									
	A	B	C							
	Norm. Vol.	Norm. Vol.	Norm. Vol.		Average	%		dev. st	err	err %
**7 dps**										
Ctrl	10865910	46781196	31104400		29583835,3	100,0		18005861,0	10395688,7	35,1
PMA	65257000	65580000	36650000		55829000,0	188,7		16610286,4	9589953,3	32,4
PMA +CAR	62257120	64574583	78960520		68597407,7	231,9		9049211,7	5224564,8	17,7
**21 dps**										
Ctrl	166063127	256054230	21535892		147884416,3	499,9		118311292,2	68307056,4	230,9
PMA	353860196	393365127	118687866		288637729,7	975,7		148500427,7	85736761,9	289,8
PMA +CAR	273065182	482298977	232222238		329195465,7	1112,8		134154949,2	77454396,1	261,8

Table of densitometry values. Densitometric quantitative analysis was carried out by using the Image Lab software version 6.1 (Bio-Rad). The pixel intensity for each band was analyzed, the background was subtracted, and the protein expression values were normalized with respect to the respective total lane of the loaded protein samples; normalized values were reported as % of control (Ctrl) at 7 dps [- https://www.bio-rad.com/it-it/applications-technologies/total-protein-normalization?ID=PODYJQRT8IG9 - https://www.bio-rad.com/webroot/web/pdf/lsr/literature/Bulletin_6360.pdf - Nature: Information for Authors & Referees > Policies > Image Integrity and Standards - Fosang AJ, Colbran RJ (2015).]

Image of WB for AIF-1 as reported in results

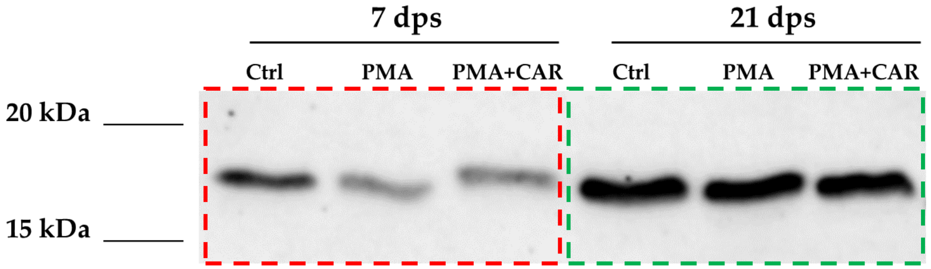

Precision Plus Protein All Blue Standards

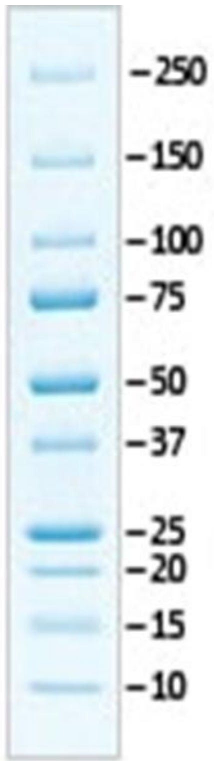

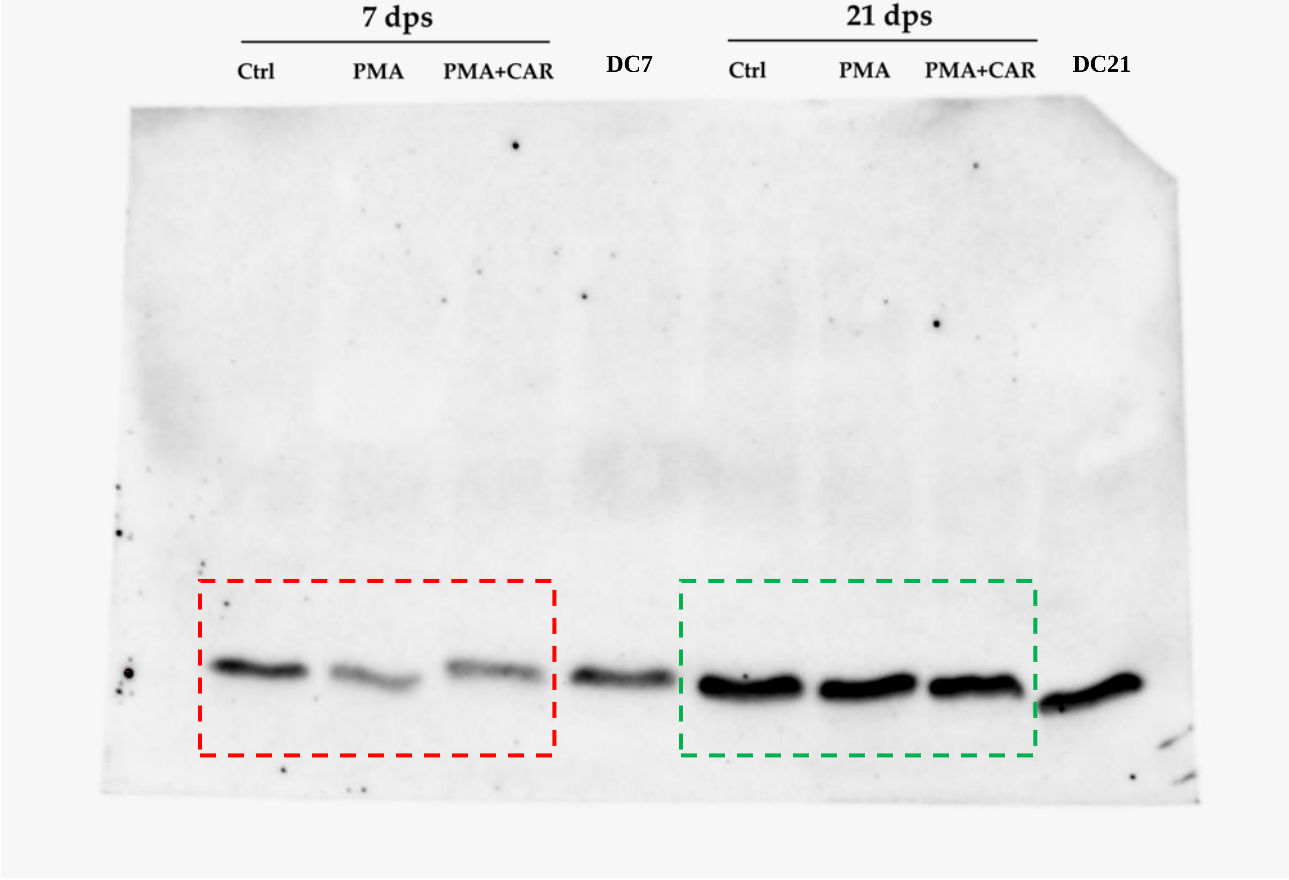

DC7/21: doubled control at 7/21 dps

20 ug protein loading per lane

Western blotting details:

Following electrophoresis on stain-free pre-cast 12% polyacrylamide gels (Bio-Rad), proteins from cell lysates were electro-transferred to polyvinylidene fluoride (PVDF) membranes. After protein transfer, the membranes were blocked for 5 minutes in EveryBlot Blocking Buffer (Bio-Rad), and then immunoblotted in EveryBlot Blocking Buffer with the following primary antibody: rabbit polyclonal anti-AIF1 primary antibody (Proteogenix, product ID A002181; dilution 1:1000). After rinsing, blots were incubated with conjugated anti-rabbit secondary antibody (Sigma-Aldrich) and the immune reactive bands were detected using an enhanced chemiluminescence method (ECL kit, Bio-Rad) with ChemiDocTM Imaging System (Bio-Rad). Densitometric quantitative analysis was carried out by using the Image Lab software version 6.1 (Bio-Rad). The pixel intensity for each band was analyzed, the background was subtracted, and the protein expression results were normalized with respect to the total lane of the loaded samples. As molecular weight markers, the Precision Plus Protein All Blue Standards (Bio-Rad, cat. no. #161-0373) was used.

Image of WB for AIF-1 as reported in results

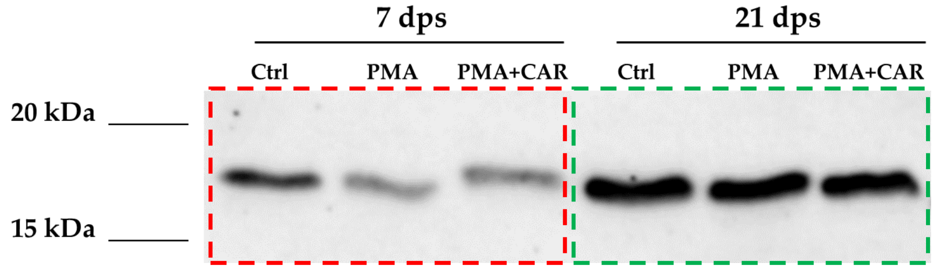

Precision Plus Protein All Blue Standards

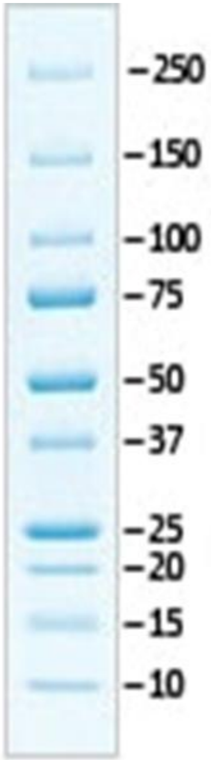

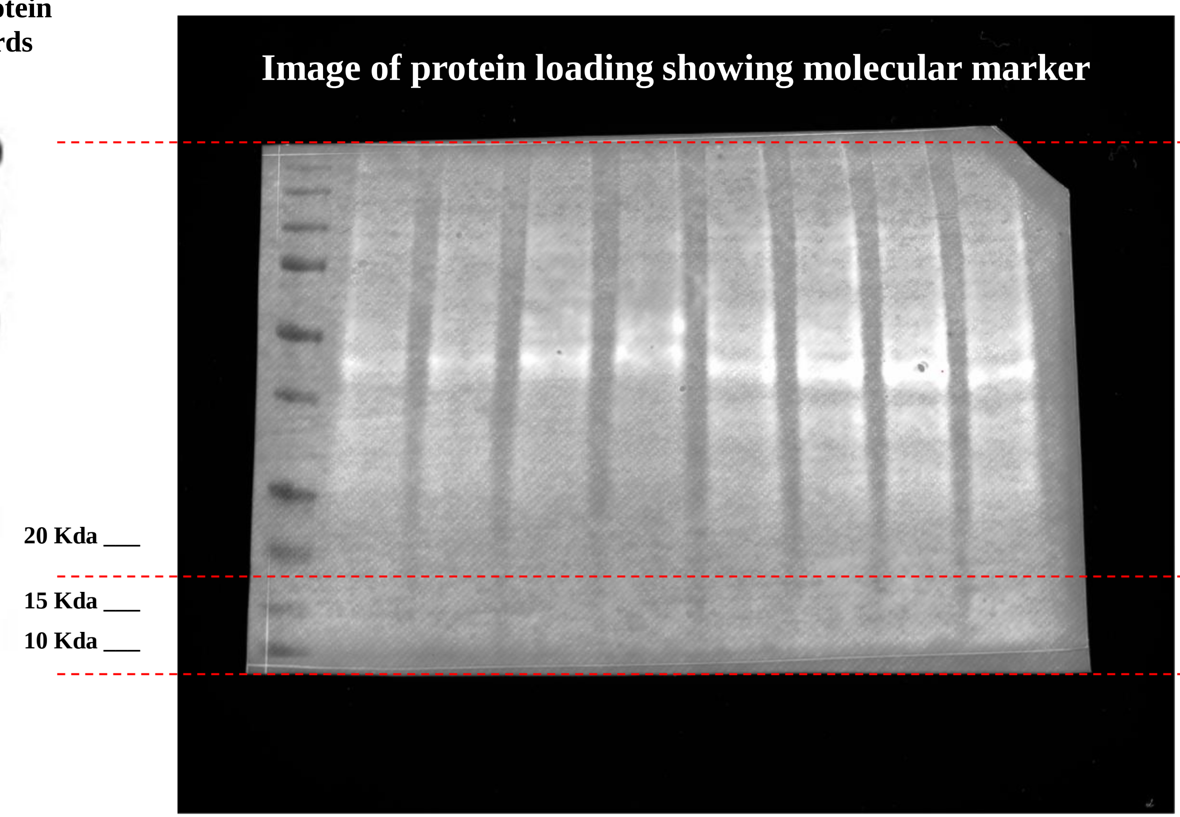

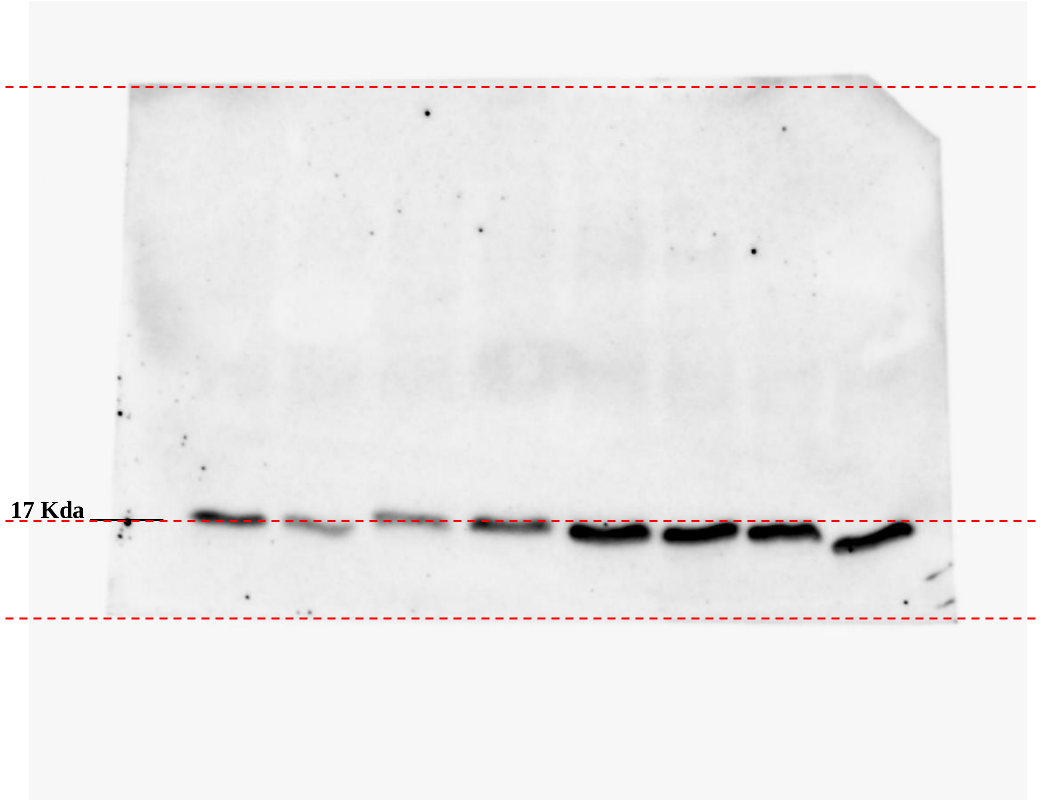

Image of WB for AIF-1 as reported in results

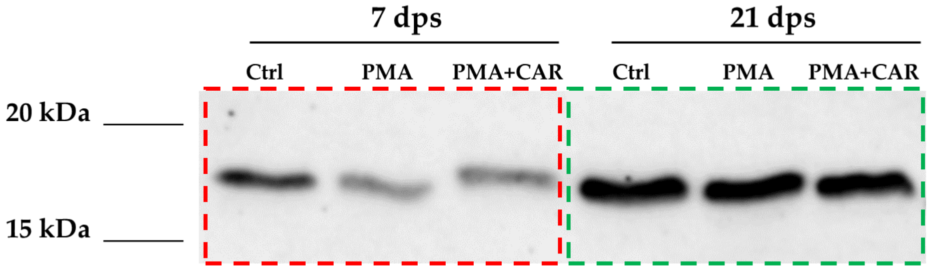

	biological replicates									
	A	B	C							
	Norm. Vol.	Norm. Vol.	Norm. Vol.		Average	%		dev. st	err	err %
**7 dps**										
Ctrl	25094090	65007920	32094090		40732033,3	100,0		21312883,5	12304999,1	30,2
PMA	40900616	32102522	31050000		34684379,3	85,2		5409080,3	3122933,9	7,7
PMA +CAR	28330075	30322316	44730075		34460822,0	84,6		8949046,0	5166734,1	12,7
**21 dps**										
Ctrl	85184768	117179487	58284768		86883007,7	213,3		29484063,4	17022631,9	41,8
PMA	55807680	71550733	53007680		60122031,0	147,6		9996070,3	5771233,9	14,2
PMA +CAR	75586038	41081353	33886038		50184476,3	123,2		22290640,2	12869507,1	31,6

Table of densitometry values. Densitometric quantitative analysis was carried out by using the Image Lab software version 6.1 (Bio-Rad). The pixel intensity for each band was analyzed, the background was subtracted, and the protein expression values were normalized with respect to the respective total lane of the loaded protein samples; normalized values were reported as % of control (Ctrl) at 7 dps [- https://www.bio-rad.com/it-it/applications-technologies/total-protein-normalization?ID=PODYJQRT8IG9 - https://www.bio-rad.com/webroot/web/pdf/lsr/literature/Bulletin_6360.pdf - Nature: Information for Authors & Referees > Policies > Image Integrity and Standards - Fosang AJ, Colbran RJ (2015).]

Beta-actin WB: Image of protein loading on membrane after transfer from stain-free gel

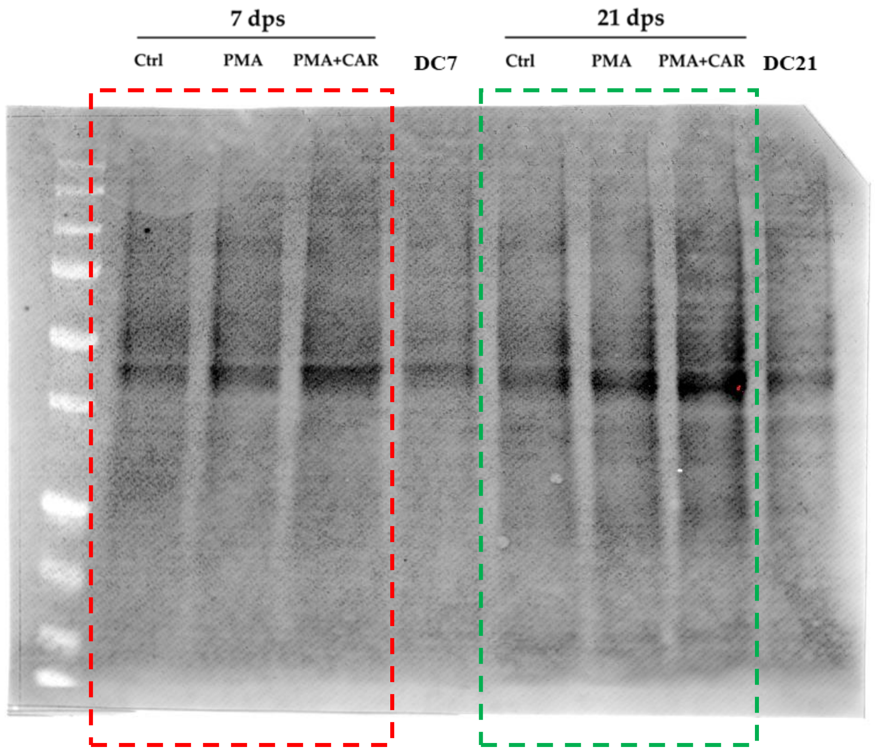

AIF-1 WB: Image of protein loading on membrane after transfer from stain-free gel

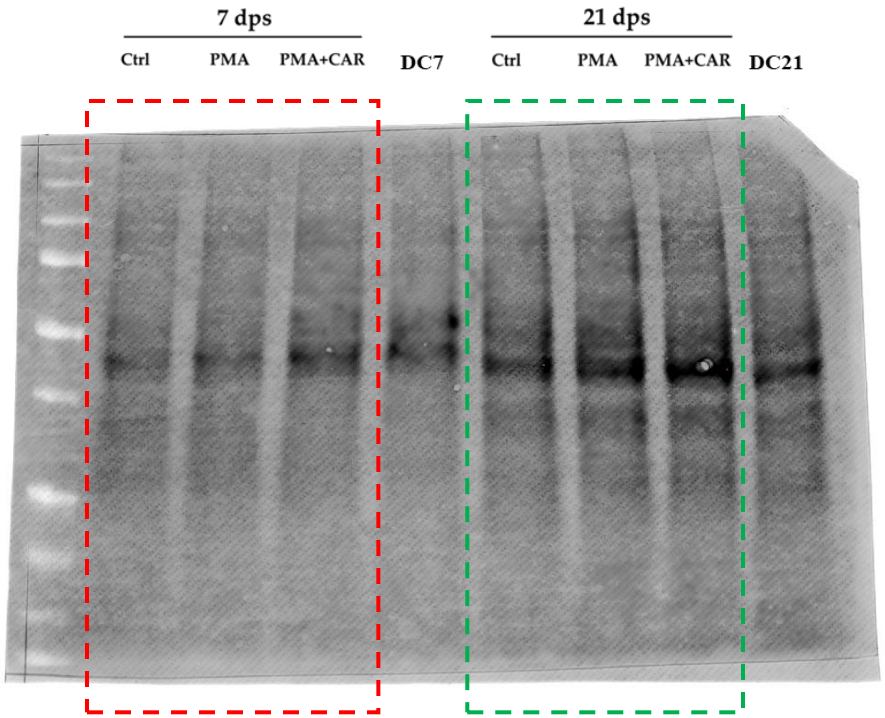

Caco-2 monolayers at 21 dps: cell viability by MTT metabolic assay

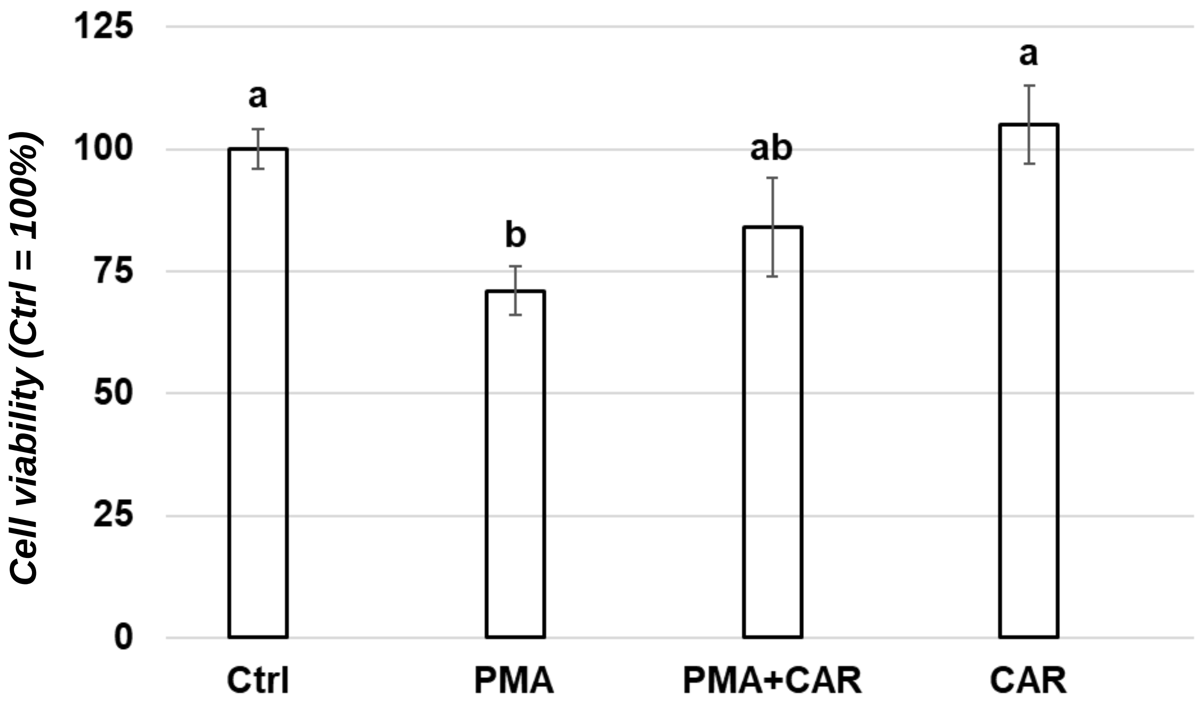

MTT viability test on Caco-2 monolayers at 21 dps (days post seeding). The histogram represents the results of viability assays by measuring metabolic activity with a standard MTT protocol performed after the experimental treatments for 48 hours (150 nM PMA, 150 nM PMA + 1 mM CAR, or 1 mM CAR alone; final concentrations in the culture medium). Data are represented as means (from n = 6 biological replicates) ± standard error of the mean (S.E.M.) and expressed as percent values with respect to the untreated control (100%). Statistical analysis: One-way ANOVA followed by Dunnett's multiple comparison test was performed using GraphPad Prism 9.4.0; different letters represent statistically different values (p value ≤ 0.05).
